# A scoping assessment of dental services at designated head and neck cancer centres in Ontario, Canada

**DOI:** 10.1186/s12903-024-03992-6

**Published:** 2024-02-13

**Authors:** Ben B. Levy, Jade Goodman, Erin Watson, Melanie Gilbert, Nick Blanas, Christopher W. Noel, Pabiththa Kamalraj, Frances C. Wright, Jonathan C. Irish, Lesley Gotlib Conn, Antoine Eskander

**Affiliations:** 1 Temerty Faculty of Medicine, University of Toronto, Toronto, Ontario Canada; 2 Dalla Lana School of Public Health, University of Toronto, Toronto, Ontario Canada; 3 Faculty of Dentistry, University of Toronto, Toronto, Ontario Canada; 4 Princess Margaret Cancer Centre, University Health Network, Toronto, Ontario Canada; 5 Sunnybrook Health Sciences Centre, 2075 Bayview Ave., Room M1 102, Toronto, Ontario M4N 3M5 Canada; 6 Department of Otolaryngology–Head & Neck Surgery/Surgical Oncology, University of Toronto, Toronto, Ontario Canada; 7 Department of Surgery, University of Toronto, Toronto, Ontario Canada; 8 Sunnybrook Research Institute, Toronto, Ontario Canada

**Keywords:** Head and neck cancer, Oncology, Dentistry, Dental services, Health equity, Health disparities, Health policy, Public health, Mixed methods

## Abstract

**Background:**

Dentists serve a crucial role in managing treatment complications for patients with head and neck cancer, including post-radiation caries and oral infection. To date, dental services for head and neck cancer patients in Ontario, Canada have not been well characterized and considerable disparities in allocation, availability, and funding are thought to exist. The current study aims to describe and assess the provision of dental services for head and neck cancer patients in Ontario.

**Methods:**

A mixed methods scoping assessment was conducted. A purposive sample of dentist-in-chiefs at each of Ontario’s 9 designated head and neck cancer centres (tertiary centres which meet provincially-set quality and safety standards) was invited to participate. Participants completed a 36-item online survey and 60-minute semi-structured interview which explored perceptions of dental services for head and neck cancer patients at their respective centres, including strengths, gaps, and inequities. If a centre did not have a dentist-in-chief, an alternative stakeholder who was knowledgeable on that centre’s dental services participated instead. Thematic analysis of the interview data was completed using a mixed deductive-inductive approach.

**Results:**

Survey questionnaires were completed at 7 of 9 designated centres. A publicly funded dental clinic was present at 5 centres, but only 2 centres provided automatic dental assessment for all patients. Survey data from 2 centres were not captured due to these centres’ lack of active dental services. Qualitative interviews were conducted at 9 of 9 designated centres and elicited 3 themes: (1) lack of financial resources; (2) heterogeneity in dentistry care provision; and (3) gaps in the continuity of care. Participants noted concerning under-resourcing and limitations/restrictions in funding for dental services across Ontario, resulting in worse health outcomes for vulnerable patients. Extensive advocacy efforts by champions of dental services who have sought to mitigate current disparities in dentistry care were also described.

**Conclusions:**

Inequities exist in the provision of dental services for head and neck cancer patients in Ontario. Data from the current study will broaden the foundation for evidence-based decision-making on the allocation and funding of dental services by government health care agencies.

**Supplementary Information:**

The online version contains supplementary material available at 10.1186/s12903-024-03992-6.

## Background

There has been a gradual increase in the number of individuals with head and neck cancers (HNCs) in Ontario, Canada (International Classification of Disease, 9th Revision [ICD-9] codes 140.3, 140.4, 141, 142.0, 142.1, 143, 144, 145, 146, 148, 161) [1]. The annual incidence of oropharynx cancer in Ontario increased by 13% from 2003 to 2010 and has been linked to human papillomavirus infection in otherwise healthy individuals [1, 2]. In 2018, the age-standardized incidence rate of cancers of the oral cavity, pharynx, and larynx in Ontario was 14.7 per 100,000, with 5-year survival ranging from 30.1% (hypopharynx cancer) to 68.4% (nasopharynx cancer) [3, 4]. Cancers of the oral cavity and pharynx represented 3.1% (male) and 1.3% (female) of all new cancer cases in Ontario in 2018, whereas cancers of larynx represented 0.8% (male) and 0.1% (female) of all new cases [3]. Treating HNCs remains a complex and multifaceted process. Large multidisciplinary teams are required to manage the various complications and side effects experienced by patients [5]. For example, radiation therapy (RT) for primary and adjuvant treatment often causes dry mouth, dental decay, and poor dentition, yielding complications such as mucositis, oral infections, post-radiation caries, and osteoradionecrosis [6–8].

Ontario Health (Cancer Care Ontario) oversees cancer services in Ontario. The Head and Neck Disease Site Group created an organizational standard for the provision of care at designated HNC centres in the province [9]. This document advises that management be carried out by a Core HNC Multidisciplinary Team responsible for assessing, treating, planning, managing, and rehabilitating patients, which includes a dentist with expertise in dental oncology [9]. Dentists serve an important role on the team in dental evaluation and stabilization of dental disease prior to RT, management of the dental complications of head and neck radiation, and long-term follow-up after the completion of RT [9]. The importance of receiving appropriate dental care throughout the continuum of HNC care to minimize morbidity has been extensively described [10–13].

Clinical practice guidelines from Canada [9, 14], the United States [15], and the United Kingdom [16, 17] have consistently outlined a standard of care which emphasizes dental services as an integral component of HNC care. Within Canada, the province of Alberta’s guideline for the ideal oral and dental care of adults with HNC offers over 40 recommendations timed before, during, and after cancer treatment; these include early referral to a dentist with expertise in dental oncology and routine post-treatment dental evaluations [14]. Alberta’s guideline was adapted from a clinical guideline created in association with the Royal College of Surgeons of England and British Society for Disability and Oral Health, which makes similar recommendations and underscores the lack of evidence for any specific pre-cancer therapy dental protocols [17].

Despite the recognized importance of maintaining optimal oral health for HNC patients, data from a 2004 cross-Canada survey revealed that only 60% of cancer centres nationwide provided all patients receiving RT to the head and neck with dental treatment prior to RT [18]. A 2023 survey of 153 HNC patients in the United States found that 23% were not asked about receiving dental care by their oncologist after the completion of RT [19]. Patients also reported challenges with both finding and paying for a dentist after HNC treatment [19]. In the United Kingdom, a retrospective survey study conducted at a hospital network found that only 52% of HNC patients who received RT saw a dentist within the year preceding their treatment [20]. This same study reported on a secondary national survey which found that one-third of HNC multidisciplinary teams across England had no access to a dentist [20].

To date, the provision of dental services for HNC patients in Ontario has not been well characterized. Because Canadians primarily receive oral health care from private dentists, considerable disparities in the access to and availability of dental services for HNC patients may exist within the province. There is also uncertainty regarding the optimal allocation and funding of dental services across Ontario’s designated HNC centres, with no recommendation provided for a minimum volume of dentists per number of treated HNC patients [9]. The current study aims to fill this gap in the literature by describing and assessing the provision of dental services for HNC patients at each of Ontario’s designated HNC centres. Understanding the current context of dental services for HNC patients within Ontario is an important first step as part of a broader needs assessment for optimizing provincial multidisciplinary and patient-centred care in HNC management.

## Methods

### Study design

A mixed methods scoping assessment was conducted between May–July 2022. Both quantitative and qualitative methods were used to garner an in-depth understanding of dental services for HNC patients. The current study employed a postpositivist paradigm, whereby the perspectives of multiple participants were compared and contrasted [21]. A mixed methods approach was adopted to allow for significance enhancement [22]. Specifically, the qualitative component of the study augmented the quantitative component by clarifying findings, exploring complementarity, and adding “real-life” examples to the results [22].

Reporting adhered to the Consolidated Criteria for Reporting Qualitative Research (COREQ) [23]. The study protocol was approved by the Research Ethics Board of Sunnybrook Health Sciences Centre (REB #5246).

### Setting and participants

The study was conducted in the province of Ontario, Canada. Dentist-in-chiefs were purposively sampled from each of Ontario’s 9 designated HNC centres, which are tertiary centres that meet provincially-set quality and safety standards for HNC care [see Additional file 1] [24]. Ontario is divided into 14 Home and Community Care Support Services organizations (formerly Local Health Integration Networks [LHINs]); from 2003 to 2010, LHINs treated between 6 and 778 cases of oral cavity cancer, and between 19 and 154 cases of larynx/hypopharynx cancer [25]. If a designated centre did not have a dentist-in-chief, an alternative stakeholder who was knowledgeable about that centre’s dental services for HNC patients participated instead. Prospective participants were identified using the principal investigator’s (A.E.) professional network. Two members of the research team (B.L., J.G.) contacted prospective participants via email and obtained written consent to participate in the study.

### Data collection

Participants first completed a 36-item survey questionnaire on the current state of dental services for HNC patients at their respective designated centre [see Additional file 2]. The survey was divided into 7 sections: demographics; billing and funding; staffing levels; workflow; frequency and setting of care; assessments and procedures; and discharge and follow-up. Survey questions were developed from a literature review and in consultation with content experts (A.E., E.W.) to ensure face and content validity [26]. Questions were piloted with a deputy dentist-in-chief at a designated HNC centre and member of the research team (E.W.). Respondent data was collected and stored on REDCap [27].

A semi-structured interview was subsequently conducted with each participant. The interview used open-ended questions to elicit insights regarding the strengths, gaps, and inequities present for dental services at each designated centre [see Additional file 3]. Questions were informed by a literature review and developed in consultation with content experts (A.E., E.W.) and an experienced medical anthropologist (L.G.C.). Findings from the survey component were used to inform/modify interview questions for this qualitative component, consistent with the “development” approach to significance enhancement in mixed methods research [22]. For example, gaps in patient care based on participants’ survey responses were explicitly probed during the interviews. The questions were piloted with E.W. Interviews were jointly conducted by B.L. (male) and J.G. (female), who at the time of the interviews were medical and dental students, respectively. The use of co-interviewing added diverse perspectives to the interviews, as well as to the thematic analysis [28]. The interviewers had no prior relationships with interviewees. Interviews were 30–60 min in length and were completed remotely via telephone or Zoom. All interviews were audio recorded.

### Data analysis

#### Quantitative data

Survey data were anonymized and reported collectively using descriptive statistics where appropriate.

#### Qualitative data

Audio recordings of the interviews were transcribed verbatim using Way With Words, a third-party transcription service. Participants were invited to review their interview transcript for accuracy. A mixed deductive-inductive approach was employed for thematic analysis of the data. For the deductive approach, the lead author (B.L.) developed a preliminary coding framework *a priori* which was informed by a literature review as well as consultations with content experts (A.E., E.W.) and a dentistry student (J.G.). For the inductive approach, a conventional content analysis was used whereby interview data was used by coders to identify new codes [29]. Two members of the research team (B.L., J.G.) coded all transcripts independently and in duplicate following completion of all interviews, irrespective of data saturation. Findings were subsequently compared to generate themes and subthemes, with consensus discussions held between coders. A qualitative descriptive approach was used to give a comprehensive and straight summary of participants’ responses [30]. NVivo 12 software was used for data management.

## Results

### Quantitative results

Of the 9 individuals contacted, 7 completed the survey (6 dentist-in-chiefs and 1 oral and maxillofacial surgeon). The remaining 2 individuals from the designated HNC centres in Hamilton and Kingston could not complete the survey due to lack of active dental services at their centres.

Table 1 presents the characteristics and workflow of dental services at 7 of Ontario’s designated HNC centres. Respondents had a median of 7 years (IQR: 5–21) of dental experience treating HNC patients, with dentists at 4 of 7 centres having completed specialized training such as a dentistry fellowship, hospital residency, and/or mentorship by dentists experienced in treating patients with HNC. While an Ontario Health Insurance Plan (OHIP)-billing dental clinic (i.e., a clinic authorized to bill the provincial health insurance plan for eligible dental services, enabling patients to access these services at no cost) was present at 5 centres, the median full-time equivalent (FTE) allotment of dentists specifically for HNC patients was 0 (IQR: 0–1.3). Respondents indicated that HNC patients are automatically assessed by a dentist at only 2 centres, with a median wait time of 6 days (IQR: 3–8) for a pre-cancer dental assessment. However, patients are provided with ongoing on-site dentistry follow-up post-radiation at 5 centres, with 6 centres able to deliver post-radiation dental care in the event of complications such as osteoradionecrosis.

**Table 1:** Characteristics and workflow of dental services at designated HNC centres in Ontario, Canada (*n* = 7)

	Frequency (%) or Median (IQR)
**Characteristics of dental services**	
Dental experience treating HNC patients^a^ (years)	7 (5–21)
Completion of specialized training prior to treating HNC patients^b^ (yes)	4/7 (57%)
Access to continuous education opportunities for treating HNC patients (yes)	3/7 (43%)
Number of dentists involved in treating HNC patients	2 (1–7)
FTE allotment of dentists for HNC patients	0 (0–1.3)
Presence of OHIP-billing dental clinic on site (yes)	5/7 (71%)
Provision of ADP- and OMRP-funded dental prostheses^c^ (yes)	5/7 (71%)
Percentage of care provided in-person (%)	99 (80–100)
**Workflow of dental services**	
Automatic assessment of HNC patients by a dentist (yes)	2/7 (29%)
Average wait time for pre-cancer dental assessment (days)^d^	6 (3–8)
Average time until discharge to community (months)^e^	12 (4–60)
Maximum number of HNC patients which a single dentist can see per day^f^	8 (6–15)
Patients provided with ongoing dentistry care/follow-up post-radiation (yes)	5/7 (71%)
Ability to deliver post-radiation dental care in the event of osteoradionecrosis or post-radiation cavities (yes)	6/7 (86%)

ADP: Assistive Devices Program; FTE: full-time equivalent; HNC: head and neck cancer; IQR: interquartile range; OHIP: Ontario Health Insurance Plan; OMRP: Oral and Maxillofacial Rehabilitation Program

^a^Response from dentist-in-chief (or equivalent)

^b^Specialized training may encompass a dentistry fellowship, hospital residency, and/or mentorship by dentists experienced in treating patients with HNC

^c^Refers to intra-oral dental prostheses; only a single centre within Ontario provides extra-oral prostheses

^d^From patient referral to first visit

^e^Excludes one designated centre at which patients are only seen in the community

^f^For the purpose of assessment, education/review, and/or treatment

**Fig. 1:**
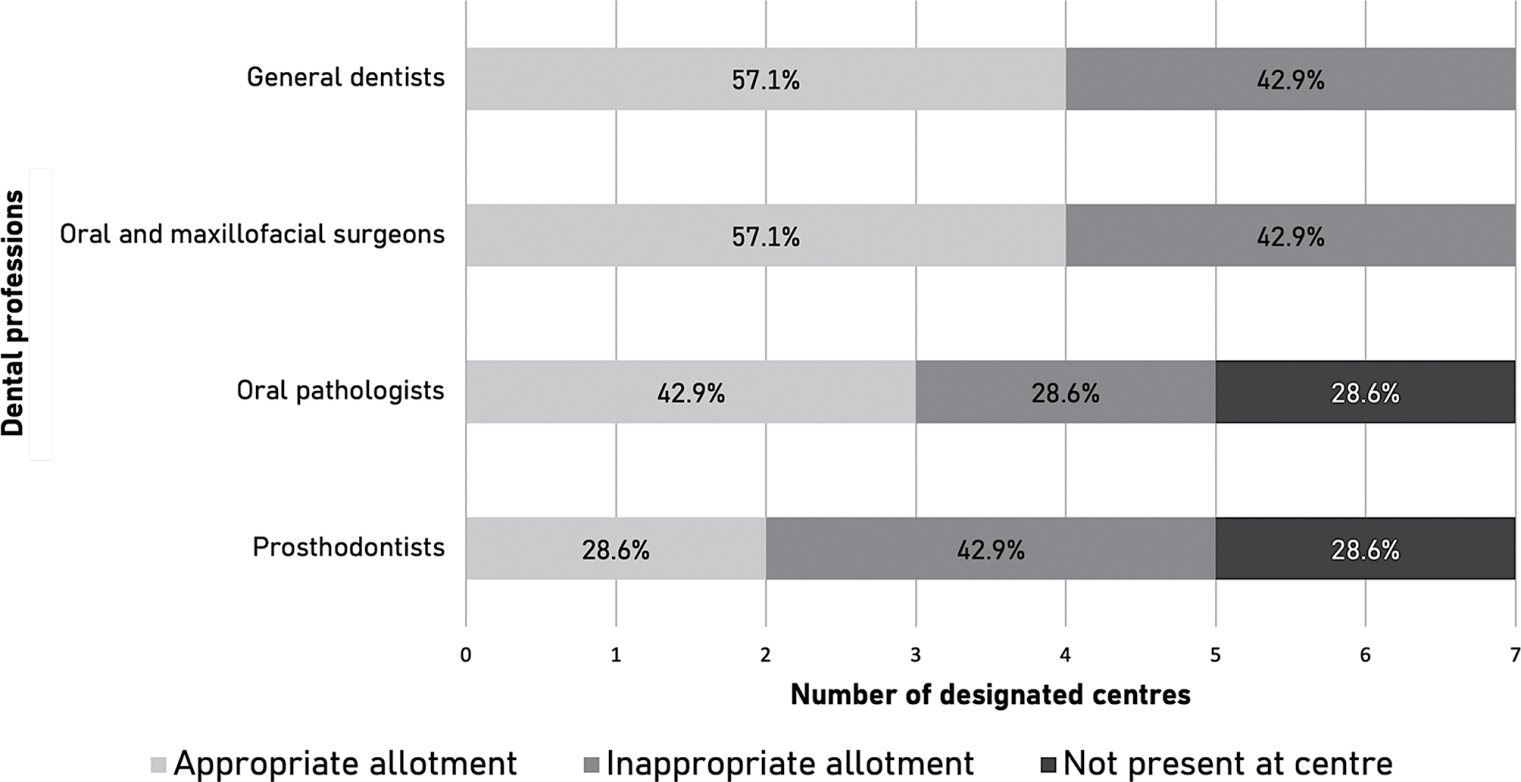
Respondents’ perceptions of the appropriateness of full-time equivalent (FTE) allotments of dental professions for HNC care at designated HNC centres in Ontario, Canada (*n* = 7)

Figure 1 presents the perceptions of respondents on the allotments of general dentists, prosthodontists, oral and maxillofacial surgeons, and oral pathologists at their respective designated HNC centres. Of the 7 respondents, 4 felt there was an appropriate FTE allotment of general dentists as well as oral and maxillofacial surgeons at their centres. These 2 professions were present at all 7 respondents’ centres. There was deemed to be an appropriate FTE allotment of oral pathologists and prosthodontists at only 3 and 2 centres, respectively. These professions were both not present at 2 of 7 centres.

### Qualitative results

A representative from each of the 9 designated HNC centres completed an interview. Of these participants, 6 were dentist-in-chiefs and 3 were knowledgeable stakeholders in other health care professional roles at designated HNC centres where no dentist-in-chief role currently exists (i.e., an oral and maxillofacial surgeon, dental hygienist, and radiation oncologist).

**Fig. 2:**
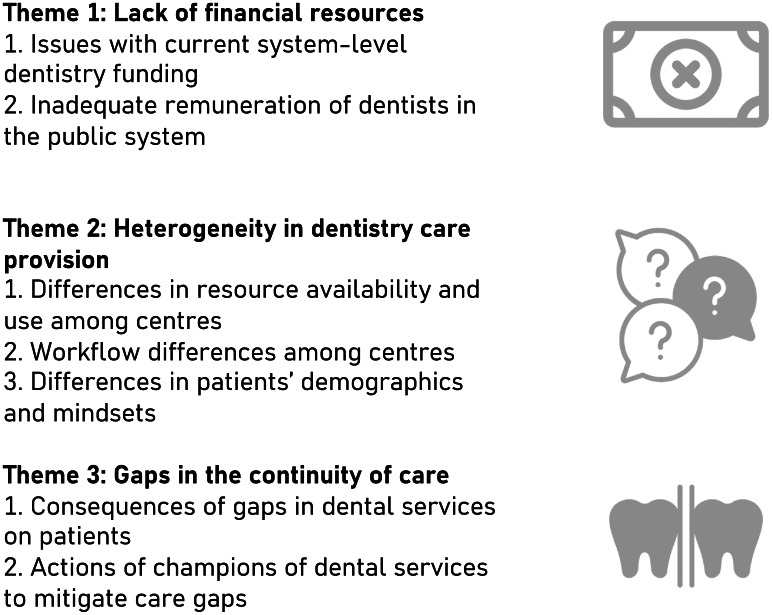
Themes and subthemes generated from interviews (*n* = 9)

Three major themes were generated from the interviews: (1) lack of financial resources; (2) heterogeneity in dentistry care provision; and (3) gaps in the continuity of care. Seven subthemes were elicited (Fig. 2).

Supporting quotations are presented in Table 2.

**Table 2:** Themes and subthemes generated from interviews (*n*=9) with supporting quotations

Theme	Subthemes	Supporting quotations
*Theme 1.* Lack of financial resources	Issues with current system-level dentistry funding	“It’s actually not legal for you as a dentist to take a tooth out without an x-ray. So, when OHIP doesn’t cover the cost of the x-ray of the tooth that you need to take out, you need to make the choice: are you going to charge the patient for the x-ray or are you not going to charge the patient and write that off as a cost?” (participant 1) “But you may have an OHIP-covered procedure, that if a dentist renders it in a hospital-based dental clinic, for a cancer patient, it would be a covered procedure. But because it’s being done in a private dental office, OHIP denies the claims. So it’s beyond the fact that there is a limitation in hospital-based resources. It also limits the patient’s ability to access OHIP-covered procedures.” (participant 5)
	Inadequate remuneration of dentists in the public system	“These patients are more medically complex, they take more time to take care of, the care is harder to render, recuperating your billing is harder to do, and you get paid less. So ultimately, you spend way more time and get paid way less money for it, as a general dentist, to do the same thing on medically complex patients, compared to healthy patients.” (participant 5) “The people who decide to do this are doing it out of interest… And you get paid, but you’re not making nearly as much as you would in the [community]. So, you need to find people who have a passion for this…. Part of it is they enjoy this type of challenge and so they’re willing to do it, but not all of their remuneration is coming from this facility.” (participant 2)
*Theme 2.* Heterogeneity in dentistry care provision	Differences in resource availability and use among centers	“It would be great if we could have an oral and maxillofacial surgeon here at least one day a week, but we don’t…. He comes maybe once every two weeks, once every three weeks, which is below what we require because of the high level of surgery.” (participant 2) “We don’t have an in-hospital clinic for dentists to be able to utilize, so we’re depending upon private offices to provide care…. We’re missing that as a modality of treatment for these patients that really require it.” (participant 5)
	Workflow differences among centers	“One of the advantages I think we have over every other dental oncology unit is, we have a 10-to-15-year-old database filled with every single patient and everything about them…. We have ready access to data and some really interesting data sets, probably that most people don’t have access to so readily.” (participant 2) “We many times have to involve communication teams, social workers, back and forth with dental offices. It’s not a well streamlined process as we would like it to be…. There are a lot of pieces outside of our control there.” (participant 9)
	Differences in patients’ demographics and mindsets	“We engage in virtual care, but our internet is not excellent across the province, especially when you go up north. So providing virtual care in an ideal world is wonderful, but it isn’t possible in a lot of our regions.” (participant 1) “I think a lot of patients don’t really understand why they need to see a dentist or what the purpose is in seeing a dentist. We’ve actually had patients in the past who’ve refused to schedule appointments because they didn’t see the purpose in it. I think, when they get their diagnosis and they realize they’re going through cancer treatments, they think teeth are the least of their concerns at that point.” (participant 4)
*Theme 3.* Gaps in the continuity of care	Consequences of gaps in dental services on patients	“Sometimes we recommend cleanings and fillings, and patients just can’t afford it. And then, that causes problems down the road in terms of losing those teeth in the future, and osteoradionecrosis of the jaw.” (participant 3) “Some patients who were not seen prior to radiation and needed extractions post-treatment, it turned out that there were complications with osteoradionecrosis. I had more than one patient say, why didn’t anybody tell me? And I’m thinking, well, unfortunately, they must have slipped through.” (participant 8) “So most of [the community dentist referral process] happens behind the scenes, but obviously, the patients also have some frustrations in terms of just being anxious about will this be covered, how timely can this be, and all of that.” (participant 9)
	Actions of champions of dental services to mitigate care gaps	“I’ve had homeless individuals come in who had to have up to 28 teeth removed, and they can’t pay for it. There are many patients [for whom] I do all the work *pro bono*.” (participant 2) “The salaries and fees that our oral surgeons would have generated when they come in, they volunteer their time to come in and work. Any billings that they generate go into a patient care fund, and that’s out of their own generosity from that oral surgery group.” (participant 6)

### Theme 1: Lack of financial resources

#### Issues with current system-level dentistry funding (“No money where our mouths are”)

Participants described system-level issues with the funding of dental services for HNC patients in Ontario, including multiple gaps in OHIP coverage. Some participants expressed frustration around the inability to bill for OHIP-covered procedures conducted in a private dental office, despite their own designated HNC centre’s lack of a hospital-based dental clinic. All participants affirmed that the current funding model accentuates inequities among vulnerable HNC patients who cannot otherwise afford dental services, as a patient’s ability to obtain coverage is dependent on their geographical proximity to a designated HNC centre with a hospital-based dental clinic.

Participants also raised issues with the fragmentation of dentistry coverage, citing an antiquated, inadequate, “surgical centric” (participant 1) OHIP fee guide which covers tooth extractions, but not the obligatory dental radiographs required prior to extractions. According to participants, the current lack of coverage for restorations, scaling, and fluoride trays, alongside other recommended treatment adjuncts, limits dentists’ ability to provide comprehensive, patient-centred care to all HNC patients and additionally restricts patient autonomy. As participant 2 describes it:

We give the patient two choices: you can either have the tooth extracted or we can do a filling on the tooth. And, if the person doesn’t have any insurance, [they can] get the tooth extracted for free or [they] would have to pay for the filling. Sometimes, probably more frequently, patients will opt to have the tooth extracted and not have the filling because [the extraction] is covered.

#### Inadequate remuneration of dentists in the public system

While the Non-Insured Health Benefits (NIHB) program does cover dental services for eligible First Nations and Inuit patients, a participant from a designated HNC centre which frequently treats Indigenous patients and refers all HNC patients out to a community dentist highlighted that some dentists choose not to accept the NIHB program because the amount reimbursed by the government is often far less than the cost of delivering treatment. Participants endorsed that other publicly funded dental care programs in Ontario, including for patients on welfare or low-income seniors, also only cover a small fraction of office overhead costs. This poses a challenge for the designated HNC centres in Ontario where the dentistry program receives no funding from its associated hospital and must instead be managed like a private practice.

Cumulatively, these gaps were described as having a negative impact on dentists’ motivations to pursue in-hospital work, where overhead costs constitute a relatively higher percentage of earned income. Participants agreed that the complexity of dentistry care for HNC patients, alongside the time and expertise required to ensure comprehensive care, are often not sufficiently compensated for general dentists in the hospital setting. Participant 1 affirms: “You’ll spend over an hour educating a patient about graft versus host disease, measuring their saliva, talking to them about trismus. All that treatment time is not remunerated by OHIP, so the minimum is covered.” While reconstructive services for HNC patients by dental specialists such as oral and maxillofacial surgeons are often covered by OHIP, they are remunerated at a reduced rate compared to private practice, further disincentivizing specialists to practice in hospital. As participant 8 – from a designated HNC centre with no dedicated dentistry program – describes it: “[We] struggled with trying to get dental people on staff, and even surgeons… Trying to get dentists over the years, just trying to get a dental component more built up. It was frustrating.”

### Theme 2: Heterogeneity in dentistry care provision

#### Differences in resource availability and use among centres

Participants’ responses revealed heterogeneity across Ontario’s designated HNC centres with respect to the current provision of dental services. Marked variation in access to dental residents and fellows, opportunities for continuing education, and collaboration with community dentists were commonly reported. Participants at centres with residents and/or fellows reported routinely conducting dental rounds, albeit with most feedback provided directly to dental trainees rather than shared between staff dentists. To date, only a single designated HNC centre in Ontario offers subspecialized post-graduate training in the care of oncology patients requiring dental care.

#### Workflow differences among centres

Several participants with an on-site dental clinic highlighted challenges with high-quality collaboration between themselves, physicians, and other multidisciplinary health care professionals, particularly if their dental clinic was physically distant from a designated HNC centre. Participants recognized the benefits of using community dentists to appropriately care for HNC patients, but also acknowledged the conceivable drawbacks of this approach, including inequities with respect to which patients would have the privilege of accessing a dental oncologist. Participant 1 says:

You’d never ask a family physician to plan somebody’s chemotherapy schedule…. So there definitely are some aspects that can be transferred over, and we’re advocating for that, but really managing some of these acute complications, it requires an expertise just like your oncologist to be able to manage.

The lack of a consistent and integrated approach to provincial-level data collection on dental services for HNC patients was also discussed by participants. While some designated HNC centres employ rigorous data collection methods and readily incorporate research into their workflow, others remain limited in their ability to evaluate dental care beyond observational data and are unable to contribute to provincial-level research and/or quality improvement initiatives. As participant 6 affirms:

That’s the other hardship about our centre, we’re not very good at data collection. So, you’ll have some centres that track everything in a beautiful database, and they can give you the numbers. And I think part of this expresses the burden of care that we’ve been trying to manage…we’re clearly anecdotal observation.

#### Differences in patients’ demographics and mindsets

The geographic location of HNC patients within Ontario was also cited as an inequity which variably affected dentistry care. Certain treatments were noted to only be available and/or subsidized at specific designated HNC centres, obligating some HNC patients to endure commutes of over 4 h or even fly in for frequent appointments. Virtual care, which participants agreed has utility in supporting comprehensive care, was said to be of limited use for patients living in Northern Ontario due to a lack of internet access in many Northern communities. Heterogeneity in patients’ understandings of dentistry care as an integral component of HNC care was also highlighted by participants, reflecting varying degrees of patient education.

### Theme 3: Gaps in the continuity of care

#### Consequences of gaps in dental services on patients

There was consensus that an inability to provide truly universal dental care to all HNC patients across Ontario has yielded long-term negative consequences for many underprivileged patients. Participants shared firsthand accounts of their own HNC patients who were unable to access necessary dental care at their designated HNC centre due to unaffordability, resulting in otherwise avoidable negative outcomes such as dental caries, loss of teeth, and/or osteoradionecrosis of the jaw. As participant 3 outlines:

We can’t start losing money to provide treatment…. [Fluoride trays and sedation] are not funded for us, so we can’t really write those costs off. So yes, definitely patients would suffer a bit from that perspective. Patients are not able to go for regular dental care. Those are the patients that we end up seeing back and managing for more serious problems in the long run, for sure.

Other examples of serious complications seen in untreated patients, such as infective endocarditis and brain and joint abscesses due to sepsis from a tooth infection, were also cited by participants. Such complications, while largely precipitated by financial barriers, were described by some participants to also be consequences of patients’ confusion and/or misunderstandings regarding the necessity of following up on dentistry-related issues.

#### Actions of champions of dental services to mitigate care gaps (“Filling in the care gaps”)

Participants characterized dentists, physicians, and other multidisciplinary health care professionals as staunch advocates for equitable dentistry care for HNC patients. While working at a non-affiliated dental clinic, participant 8 would often encounter HNC survivors who were burdened by lingering dental issues due to a lack of education and consistent follow-up. As participant 8 describes it:

Nobody told [the HNC survivors] about all this dry mouth and all these side effects from the cancer treatment. So, I called up [designated HNC centre] to try to speak to somebody there, just to see what the protocol was there for cancer patients. And they said, unfortunately, they didn’t really have anything in place. [HNC survivors] were just sent back to their dentist, and if they didn’t have a dentist, they were just lost and were deterred.

Nearly all participants reported either personally doing *pro bono* dental work for HNC patients or knowing colleagues who did. At several designated HNC centres with no in-hospital dental clinic, underprivileged HNC patients were said to be entirely dependent on the charity of community dentists willing to provide free dental care, but who “want to remain anonymous because they don’t want people to take advantage of them” (participant 7). One centre’s oral and maxillofacial surgeons altruistically donate all their in-hospital earnings to a designated patient care fund to cover uninsured dental services for low-income HNC patients. With respect to this arrangement, participant 6 argues: “That’s not really an appropriate way to fund health care – through our oral surgeons’ generosity in not accepting the billings for the work that they’ve done. It shouldn’t be that way.”

## Discussion

There has been extensive discussion on the importance of providing appropriate dental services for HNC patients. Poor oral hygiene has been shown to be a possible prognostic factor of HNC [10], with dental evaluation consistently cited as a key factor in optimizing HNC treatment and minimizing complications [11, 31, 32]. The current study adds to the evidence base on the consequences of inadequate structure of and access to dental services for HNC patients. Our results highlight stark inequities in the delivery of dental services to HNC patients throughout Ontario, including instances where patients’ geographical proximity to a designated HNC centre is the limiting factor in care provision. Participants’ personal experiences illustrate the devastating reality for vulnerable HNC patients in Ontario, who represent a critical segment of the HNC patient population which does not currently receive appropriate and timely dental care.

Participants in the current study reported difficulty with recruiting and retaining dentists and dental specialists within the in-hospital setting. This supports previous research showing that Canadian dentists may take issue with public plans and/or purposefully decide to reduce the amount of public insurance within their private practices [33]. Several participants noted the importance of involving community dentists in the longitudinal management of HNC patients following discharge, which aligns with previous literature on this topic from the United Kingdom [34]. However, evidence from the United States suggests post-graduate general dentistry residency programs are experiencing challenges to incorporating oncology curricula, including bone marrow transplantation, cancer biology, immunotherapy, and prosthetics for use during head and/or neck surgery [35]. Moreover, obligating centres without a dental clinic to refer all HNC patients to community dentists for treatment does create fundamental inequities between patients. In a 2007 survey from the United States, 56% of 16 responding National Cancer Institute-designated comprehensive cancer centres had no dental department and no standardized protocols for oral treatment complications [36]. This finding remains comparable to Ontario’s situation over 15 years later, as 4 of Ontario’s 9 designated HNC centres (44%) still do not have an on-site dental clinic. Notably, it was announced in November 2022 that Medicare in the United States will begin funding dental services “integral to treating a beneficiary’s medical condition,” including prior to treatment for HNC, in 2024 [37]. Recent announcement of the Canada Dental Benefit is a promising step toward comprehensive dental coverage for Canadians, but coverage for all members of households with an income of less than $90,000 CAD is not anticipated to be available until 2025 [38]. Even then, reimbursement payments may not cover the full spectrum of dental services necessary for comprehensive HNC care.

Our analysis provides a foundation for 3 recommendations which represent the first and necessary steps toward addressing these challenges. First, a comprehensive review of the current OHIP fee guide for dental services should be undertaken with the goal of enhancing and modernizing public coverage. Due consideration should be given to adding coverage for essential non-surgical dental services in the continuum of HNC care, including dental radiographs, fluoride trays, and restorations. A comprehensive fee guide should also explicitly fund patient education delivered by providers, such that HNC patients understand the complexities of their condition as well as the importance of dental follow-up.

Second, funding should be designated for the implementation of a fully resourced dental oncology unit, including an on-site dental clinic, at all designated HNC centres in Ontario. Fully delivering comprehensive HNC care at a single site will enhance accessibility, especially if patients are geographically distant from tertiary care infrastructure. It will also serve to reduce fragmentation and streamline communication/coordination between members of a Core HNC Multidisciplinary Team, thereby enhancing continuity of care. A minimum FTE allotment for dentists who specifically treat HNC patients should be designated and funded at each centre. As an interval step until implementation, private dental offices which provide care to HNC patients should be permitted to bill OHIP for essential dental services in the care of HNC.

Third, data collection on dental outcomes for HNC patients should be prioritized. A standardized list of outcomes of interest, which may include incidences of dental complications/side effects, should be developed specifically for designated HNC centres to track. Investments in infrastructure to support data integration across designated HNC centres would also help facilitate future province-wide research and quality improvement initiatives for dental services.

This study is the first to characterize the provision of dental services for HNC patients in Ontario by synthesizing data from each of the province’s 9 designated HNC centres. These data will broaden the foundation for evidence-based decision-making on the allocation and funding of dental services for HNC patients by provincial health care agencies such as Ontario Health (Cancer Care Ontario). Future studies which analyze the association between access to dental services for HNC patients and health-system level outcomes (e.g., emergency department visits) may help strengthen the case for additional investments in dental services. Directly involving patient advocates in advocacy work may also help policymakers gain a better understanding of the inequities affecting care.

## Limitations

We acknowledge several study limitations. The current study sought to examine dental services at a systems level and thus reported on the perspectives of HNC patients via proxy only. While this added to the potential for bias, there was consensus among participants regarding the frustrations of patients and disparities in care which have negatively impacted patients. Future studies which incorporate input from additional key stakeholders, including HNC patients and/or cancer centre leadership, will be important to provide a more comprehensive perspective on the delivery of dental services. It was also necessary to include several non-dentist medical professionals as participants, and participants in Hamilton and Kingston were unable to complete the survey component of the study since most questions addressed elements of an active dentistry program. Nevertheless, even participants without an active dentistry program were highly knowledgeable regarding dental services for HNC patients at their designated HNC centre.

## Conclusions

Overall, across Ontario’s designated HNC centres, dental services for HNC patients are concerningly under-resourced. Marked disparities exist in the provision of dental services for HNC patients and depend on which specific designated HNC centre care a patient is treated at. Continued resource insufficiency and underfunding are likely precipitating worse health outcomes for vulnerable HNC patients, which is an area of exploration for future studies.

### Electronic supplementary material

Below is the link to the electronic supplementary material.

**Supplementary Material 1: Additional file 1**. List of Ontario’s designated head and neck cancer centres

**Supplementary Material 2: Additional file 2**. Survey questionnaire

**Supplementary Material 3: Additional file 3**. Interview guide

## Data Availability

The datasets used and/or analyzed during the current study are available from the corresponding author on reasonable request.

## Abbreviations

FTE: full-time equivalent
HNC: head and neck cancer
IQR: interquartile range
NIHB: Non-Insured Health Benefits
OHIP: Ontario Health Insurance Plan
RT: radiation therapy
