# Supplementary material for: A scoping assessment of dental services at designated head and neck cancer centres in Ontario, Canada
Source: BMC Oral Health. 2024 Feb 13;24:232. doi: 10.1186/s12903-024-03992-6 (PMC10865540; doi:10.1186/s12903-024-03992-6)
Supplement: Supplementary file 1 — Supplementary Material 1: Additional file 1. List of Ontario’s designated head and neck cancer centres [file 12903_2024_3992_MOESM1_ESM.docx]

**Ontario’s Designated Head and Neck Cancer Centers**

1. Cancer Centre of Southeastern Ontario (Kingston)
2. Northeast Cancer Centre (Sudbury)
3. Odette Cancer Centre (Toronto)
4. Princess Margaret Cancer Centre (Toronto)
5. The Ottawa Hospital Cancer Centre (Ottawa)
6. Juravinski Cancer Centre (Hamilton)
7. London Regional Cancer Program (London)
8. Regional Cancer Care Northwest (Thunder Bay)^a^
9. Windsor Regional Cancer Centre (Windsor)^a^

^a^Partner centers which deliver chemotherapy and radiation, but not surgery
