# Supplementary material for: A scoping assessment of dental services at designated head and neck cancer centres in Ontario, Canada
Source: BMC Oral Health. 2024 Feb 13;24:232. doi: 10.1186/s12903-024-03992-6 (PMC10865540; doi:10.1186/s12903-024-03992-6)
Supplement: Supplementary file 2 — Supplementary Material 2: Additional file 2. Survey questionnaire [file 12903_2024_3992_MOESM2_ESM.pdf]

Scoping Assessment - Dentistry Survey

Thank you for agreeing to participate in our study, "A Scoping Assessment of Dentistry and Speech-Language Pathology Care at Head and Neck Cancer Designated Centres in Ontario, Canada".

This study is an exploration of the current landscape of dentistry and SLP care available for head and neck cancer patients in Ontario.

Each participant will be interviewed for approximately 45-60 minutes by 1-2 members of the research team. You will schedule an interview date and time with a member of the research team. Prior to your interview, you are asked to complete this short survey which will provide additional context for your interview and assist in the exploration of dentistry care for head and neck cancer patients at your head and neck cancer designated centre.

This survey is expected to take 10-15 minutes to complete. Some questions will ask you for a number or percentage value. For these questions, if you do not know (or cannot find) an exact quantity, please provide your best estimate. If you wish to elaborate on a response, all text boxes will accept free-text responses.

What is your participant ID?

____
(You were provided with your participant ID via email.)

Which head and neck cancer designated centre in Ontario do you presently work at?

- ◯ London Health Sciences Centre
- ◯ St. Joseph's Healthcare Hamilton, Charlton Campus
- ◯ University Health Network
- ◯ Sunnybrook Health Sciences Centre
- ◯ Kingston General Hospital
- ◯ The Ottawa Hospital
- ◯ Health Sciences North/Horizon Santé-Nord
- ◯ Thunder Bay Regional Health Sciences Centre
- ◯ Windsor Regional Hospital

How many years of experience do you have working as a dentist with head and neck cancer patients?

____

Do dentists at [designated_centre_v2] complete any specialized training prior to treating head and neck cancer patients?

- ◯ Yes (please describe)
- ◯ No

Please describe the specialized training completed by dentists at [designated_centre_v2]:

____

Do dentists at [designated_centre_v2] currently have access to any continuous education/educational opportunities for treating head and neck cancer patients?

- ◯ Yes (please describe)
- ◯ No

Please describe the continuous education/educational opportunities which dentists have access to at [designated_centre_v2]:

____

The next three (3) questions pertain to dentistry billing and funding at [designated_centre_v2].

Is there an Ontario Health Insurance Plan (OHIP)-billing dental clinic on site at [designated_centre_v2]?	◯ Yes<br>◯ No<br>◯ Other (please explain)
Other (please explain):	__________________________________
Is [designated_centre_v2] able to provide Assistive Devices Program (ADP)-funded dental prostheses?	◯ Yes, intra-oral prostheses only<br>◯ Yes, extra-oral prostheses only<br>◯ Yes, both intra- and extra-oral prostheses<br>◯ No
Is [designated_centre_v2] able to provide Oral and Maxillofacial Rehabilitation Program (OMRP)-funded dental prostheses?	◯ Yes, intra-oral prostheses only<br>◯ Yes, extra-oral prostheses only<br>◯ Yes, both intra- and extra-oral prostheses<br>◯ No

The next seven (7) questions pertain to the staffing levels of dentists at [designated_centre_v2].

How many dentists at [designated_centre_v2] are involved in the care of head and neck cancer patients? __________________________________

What is the full-time equivalent (FTE) allotment of dentists specifically for head and neck cancer patients at [designated_centre_v2]? __________________________________

Does the FTE allotment of dentists available at [designated_centre_v2] ever impact decisions to refer head and neck cancer patients to dentistry?

- ◯ Yes
- ◯ No

In your opinion, does [designated_centre_v2] currently have an appropriate FTE allotment of dentists for optimal head and neck cancer patient care?

- ◯ Yes
- ◯ No

In your opinion, does [designated_centre_v2] currently have an appropriate FTE allotment of prosthodontists for optimal head and neck cancer patient care?

- ◯ Yes
- ◯ No
- ◯ We do not have any prosthodontists

In your opinion, does [designated_centre_v2] currently have an appropriate FTE allotment of oral and maxillofacial surgeons for optimal head and neck cancer patient care?

- ◯ Yes
- ◯ No
- ◯ We do not have any oral and maxillofacial surgeons

In your opinion, does [designated_centre_v2] currently have an appropriate FTE allotment of oral pathologists for optimal head and neck cancer patient care?

- ◯ Yes
- ◯ No
- ◯ We do not have any oral pathologists

The next 10 questions pertain to workflow as a dentist at [designated_centre_v2].

We want to understand how you allocate your time between your primary areas of responsibility at work. Using percentages, specify how your time is divided between each of your primary areas of responsibility.<br><br>Please ensure your percentages add up to 100%.	__________________________________<br>(Sample response: 40% seeing new patients, 30% administrative tasks, etc.)
What percentage of head and neck cancer patients receiving radiation at [designated_centre_v2] are referred to a dentist at [designated_centre_v2]?	__________________________________
What percentage of head and neck cancer patients receiving surgery at [designated_centre_v2] are referred to a dentist at [designated_centre_v2]?	__________________________________
On average, what number of head and neck cancer patients are seen per week by one (1) dentist at [designated_centre_v2]?	__________________________________
What is the maximum number of head and neck cancer patients which can be seen in one (1) day by one (1) dentist at [designated_centre_v2]?	__________________________________
In your opinion, are dentists at [designated_centre_v2] currently seeing an appropriate number of head and neck cancer patients for optimal patient care?	◯ Yes<br>◯ No
Do you believe dentistry at [designated_centre_v2] is overworked, underworked, or neither?	◯ Overworked<br>◯ Underworked<br>◯ Neither
Are head and neck cancer patients automatically assessed by a dentist at [designated_centre_v2]?	◯ Yes<br>◯ No (please specify below)
What triggers the assessment of a head and neck cancer patient by dentistry?	__________________________________
What is the average wait time for a pre-cancer treatment dental assessment for a head and neck cancer patient from patient referral to first visit at [designated_centre_v2]?	__________________________________<br>(Please be sure to specify time units in your answer (i.e., X days/weeks/months).)

The next (5) questions pertain to the frequency of dental care and the care setting at [designated_centre_v2].

On average, once intake for dentistry is completed, how frequently are head and neck cancer inpatients seen by a dentist at [designated_centre_v2]?<br><br>If dentists do not see inpatients at your centre, please input "N/A".	__________________________________<br>(Please be sure to specify time units in your answer (i.e., every X days/weeks/months).)
In your opinion, are dentists at [designated_centre_v2] currently seeing head and neck cancer inpatients at an appropriate frequency for optimal patient care?	◯ Yes<br>◯ No<br>◯ Dentists at our centre do not see inpatients
On average, once intake for dentistry is completed, how frequently are head and neck cancer outpatients seen by a dentist at [designated_centre_v2]?	__________________________________<br>(Please be sure to specify time units in your answer (i.e., every X days/weeks/months).)
In your opinion, are dentists at [designated_centre_v2] currently seeing head and neck cancer outpatients at an appropriate frequency for optimal patient care?	◯ Yes<br>◯ No<br>◯ Dentists at our centre do not see outpatients
What percentage of dentistry care for head and neck cancer patients at [designated_centre_v2] is currently provided in-person, as opposed to virtually or via telephone?	__________________________________

The next four (4) questions pertain to assessments and procedures completed by dentists at [designated_centre_v2].

What are the most common assessments completed by dentists for head and neck cancer patients at [designated_centre_v2]?<br><br>Please enter one (1) assessment per row.	__________________________________<br>(Please enter one (1) assessment per row.)
What number of<br><br>[common_assessments_v2]<br><br>were completed by dentists at [designated_centre_v2] in the past 12 months?<br><br>Please enter one (1) number per row, corresponding to the order of your answers in the previous question.	__________________________________<br>(Please enter one (1) number per row, corresponding to the order of your answers in the previous question.)
What are the most common procedures completed by dentists for head and neck cancer patients at [designated_centre_v2]?<br><br>Please enter one (1) procedure per row.	__________________________________<br>(Please enter one (1) procedure per row.)
What number of<br><br>[common_procedures_v2]<br><br>were completed by dentists in the past 12 months at [designated_centre]?<br><br>Please enter one (1) number per row, corresponding to the order of your answers in the previous question.	__________________________________<br>(Please enter one (1) number per row, corresponding to the order of your answers in the previous question.)

The final three (3) questions pertain to follow-up care and discharge at [designated_centre_v2].

If a patient were to develop osteoradionecrosis or post-radiation cavities, could post-radiation dental care be delivered at [designated_centre_v2]?	◯ Yes<br>◯ No<br>◯ Other (please explain)
Other (please explain):	__________________________
Are head and neck cancer patients at [designated_centre_v2] provided ongoing dental care/follow-up post-radiation?	◯ Yes<br>◯ No<br>◯ Other (please explain)
Other (please explain):	__________________________
On average, after what period of time are head and neck cancer patients receiving dental care at [designated_centre_v2] discharged to the community?	__________________________<br>(Please be sure to specify time units in your answer (i.e., after X days/weeks/months/years).)
