# Supplementary material for: A scoping assessment of dental services at designated head and neck cancer centres in Ontario, Canada
Source: BMC Oral Health. 2024 Feb 13;24:232. doi: 10.1186/s12903-024-03992-6 (PMC10865540; doi:10.1186/s12903-024-03992-6)
Supplement: Supplementary file 3 — Supplementary Material 3: Additional file 3. Interview guide [file 12903_2024_3992_MOESM3_ESM.docx]

**Questions for dentist-in-chief (or equivalent):**

1. Can you tell me what it’s like to provide dentistry care for head and neck cancer patients at your centre?
   - When you say it’s X, what makes it X?
   - Has it always been X?

*Current state of dentistry care (probe about how and why throughout):*

1. At your centre, what do you believe are the current **gaps** in the management of head and neck cancer patients with respect to dentistry care?
   - What actions do you think should be prioritized at your centre to help resolve these gaps?
2. At your centre, what do you believe are the current **inequities** impacting the provision of dentistry care to head and neck cancer patients?
   - What differences exist between the care patients receive at your centre versus other centres in the province?
3. At your centre, what do you believe are currently the greatest **strengths** of dentistry care?
4. At your centre, where do you believe there is currently the most **room for improvement** for dentistry care?
   - What actions would you take to improve the quality of dentistry care for head and neck cancer patients at your centre?

*Staffing levels and expertise of dentists for head and neck cancer patients:*

1. What do you believe is contributing to the (availability/lack of availability) of dentists for head and neck cancer patients at your centre?
   - **(If lack of availability)** What do you think would be required for more dentists to be available for head and neck cancer patients at your centre?
2. Can you think of any additional areas of training in caring for head and neck cancer patients which dentists at your centre would benefit from?
   - Why should these areas of training be prioritized?

*Dentist workflow:*

1. Are there any duties/responsibilities you believe you should be spending **more** time on *(if yes, explain)*? What prevents you from spending more time on these duties/responsibilities?
   - Are there any duties/responsibilities you believe you should be spending **less** time on *(if yes, explain)*? What are prevents you from spending less time on these duties/responsibilities?
2. Can you describe how you collaborate with prosthodontists, oral and maxillofacial surgeons, and/or oral pathologists?
   - How important is it for you to be able to collaborate with these specialists?
   - At your centre, what opportunities are in place for **receiving feedback** about your treatment plans?
   - Does the dental service have rounds? Do dentists attend multidisciplinary rounds?
   - Can you give an example?
3. Can you describe (why you/why you do not) believe your centre is providing **“optimal” care** in terms of:
   - The number of patients which can be seen by dentists
   - The frequency with which (inpatients and outpatients) can be seen by dentists
     - (Probe about facilitators and barriers to optimal number/frequency)
4. **Why** do you believe that dentistry at your centre is (overworked/underworked/neither)?

*Changes/evolution to dentistry at the head and neck cancer designated centre:*

1. Specific to your centre, describe any major **staffing changes** with respect to dentistry which have happened, as far back as you can remember.
   - Were there any changes you felt were **most** helpful? Why?
   - Were there any changes you felt were **not** helpful? Why?
2. Specific to your centre, describe any major changes to **procedures/patient care** with respect to dentistry (e.g., due to changes in evidence base), as far back as you can remember.
   - Were there any changes you felt were **most** helpful? Why?
   - Were there any changes you felt were **not** helpful? Why?
3. Specific to your centre, how has **funding** impacted the provision of dentistry care to head and neck cancer patients?
4. Specific to your centre, can you describe how **billing** operates with respect to the provision of dentistry care to head and neck cancer patients?
   - What procedures do you specifically bill for?
   - Can you describe which dental services are **not** billed for but are still necessary for the provision of dentistry care?
